# Human Milk From Atopic Mothers Has Lower Levels of Short Chain Fatty Acids

**DOI:** 10.3389/fimmu.2020.01427

**Published:** 2020-07-21

**Authors:** Lisa F. Stinson, Melvin C. L. Gay, Petya T. Koleva, Merete Eggesbø, Christine C. Johnson, Ganesa Wegienka, Elloise du Toit, Naoki Shimojo, Daniel Munblit, Dianne E. Campbell, Susan L. Prescott, Donna T. Geddes, Anita L. Kozyrskyj

**Affiliations:** ^1^School of Molecular Sciences, University of Western Australia, Perth, WA, Australia; ^2^inVIVO Planetary Health of the Worldwide Universities Network (WUN), West New York, NJ, United States; ^3^Department of Pediatrics, University of Alberta, Edmonton, AB, Canada; ^4^Department of Environmental Exposure and Epidemiology, Norwegian Institute of Public Health, Oslo, Norway; ^5^Department of Public Health Sciences, Henry Ford Hospital, Detroit, MI, United States; ^6^Division of Medical Microbiology, University of Cape Town, Cape Town, South Africa; ^7^Department of Pediatrics, Chiba University, Chiba, Japan; ^8^Department of Paediatrics and Paediatric Infectious Diseases, Institute of Child Health, Sechenov First Moscow State Medical University (Sechenov University), Moscow, Russia; ^9^Inflammation, Repair and Development Section, National Heart & Lung Institute, Imperial College London, London, United Kingdom; ^10^Department of Allergy and Immunology, Children's Hospital at Westmead, University of Sydney, Sydney, NSW, Australia; ^11^The ORIGINS Project, Telethon Kids Institute, University of Western Australia, Perth, WA, Australia

**Keywords:** human milk, short chain fatty acids, atopy, allergy, international cohort, breast milk

## Abstract

Short chain fatty acids (SFCAs) are microbial metabolites produced in the gut upon fermentation of dietary fiber. These metabolites interact with the host immune system and can elicit epigenetic effects. There is evidence to suggest that SCFAs may play a role in the developmental programming of immune disorders and obesity, though evidence in humans remains sparse. Here we have quantified human milk (HM) SCFA levels in an international cohort of atopic and non-atopic mothers (*n* = 109). Our results demonstrate that human milk contains detectable levels of the SCFAs acetate, butyrate, and formate. Samples from atopic mothers had significantly lower concentrations of acetate and butyrate than those of non-atopic mothers. HM SCFA levels in atopic and non-atopic women also varied based on maternal country of residence (Australia, Japan, Norway, South Africa, USA). Reduced exposure to HM SCFA in early life may program atopy or overweight risk in breastfed infants.

## Introduction

Human milk (HM) confers numerous benefits to the developing infant, an effect attributed to its many bioactive metabolites. The evidence for some of the long-term health benefits of HM is inconclusive (1). Regarding the prevention of atopic diseases through breastfeeding, this varies across countries and in particular, according to the atopic phenotype of the mother (2). While genetics and epigenetics play a role in the inheritance of atopic disease (3, 4), the role of HM metabolites remains underexplored in this field. Still in its infancy, the study of the HM metabolome has proven valuable in identifying variability by maternal phenotype, diet, and disease state (5, 6). Short chain fatty acids (SCFAs) are key metabolites of microbial fermentation of fiber that have links with host health. Early-life exposure to SCFAs has been shown to protect against atopy (7). When administered to pregnant mice, the SCFA acetate has prevented offspring from developing atopic airway inflammation (8). These findings are corroborated by human data of associations between high maternal serum acetate levels during pregnancy and decreased risk of respiratory symptoms in young infants (8). Similarly, propionate has been shown to protect against allergic airway disease in mice via its effects on dendritic cell biology (9), while butyrate induces the differentiation of colonic regulatory T cells (10). Further, murine studies have demonstrated that prebiotic fiber supplementation during pregnancy or lactation reduces risk of atopy in offspring (11, 12). Similar trials are currently underway in humans [SYMBA (13) and PREGRALL (14)]. Recently, Lee-Sarwar et al. reported higher fecal acetate levels (relative to total SCFA) in pregnant women of children less likely to develop atopic disease (15).

SCFAs (formate, acetate, propionate, butyrate, and valerate) are intermediate and end products of dietary carbohydrate fermentation by gut bacteria (7). These microbial metabolites are concentrated in the colon and some are distributed systemically after absorption (8, 9, 16). Through their interaction with G-protein-coupled receptors and their inhibition of histone deacetylases, SCFAs are able to elicit a broad range of biological effects, including promotion of regulatory T cell responses and tolerance, mucus secretion and epithelial barrier integrity in the gut, and synthesis of bone marrow dendritic cell precursors (9, 17, 18). A broad range of bacteria are also present in HM (19). HM SCFAs are likely produced by the maternal gut microbiota and distributed to the mammary gland via the circulation. They may also be produced by the resident HM microbiota; however, evidence for this possibility is currently lacking. To date, there has been limited investigation into HM SCFA profiles. Smilowitz et al. were the first to document the presence of acetate and butyrate in HM samples collected 90 days postpartum, finding that these SCFAs were highly variable among women (20). In HM samples from a single woman, acetate, butyrate and formate were detected as early as 24 days postpartum (21). All three of these SCFAs were identified by nuclear magnetic resonance (NMR) at 1-2 months postpartum in a larger study of women (22). Butyrate has also been documented in studies of HM fat or fatty acids (23, 24). Meng et al. reported the presence of acetate and butyrate in HM from women with and without irritable bowel disease, finding higher acetate levels in women treated with aminosalicylates (25). Finally, Gómez-Gallego et al. performed NMR metabolic profiling of 79 HM samples from 4 international cohorts. They identified acetate, butyrate, and formate in these samples and reported differences in acetate and formate levels between countries (26).

Total SCFA levels are elevated in the stool of lactating women at 1 month postpartum compared to non-pregnant women (27), implicating their importance to the nursing infant. HM SCFAs have been shown experimentally to prevent atopic disease, but breastfeeding by atopic mothers does not protect against atopy to the same extent as breastfeeding by non-atopic mothers (2). This discrepancy may be a function of reduced levels of SCFAs in HM among atopic mothers, though this has not been tested. Herein, we profiled SCFA levels in HM samples from atopic and non-atopic mothers from six international sites, including two countries with high rates of atopic disease. We hypothesized that atopic women would exhibit reduced levels of HM SCFAs.

## Methodology

### Study Design

In this descriptive study, 109 HM samples from 6 cohort studies from different countries were analyzed (5). The cohorts were from Perth, Australia (*n* = 29 from 2 cohorts); Chiba, Japan (*n* = 12); Detroit, USA (*n* = 18); Oslo, Norway (*n* = 40); Cape Town, South Africa (*n* = 10). These cohorts were sampled across countries to identify women with and without atopic disease. Whenever possible, samples were obtained from women who delivered vaginally and did not receive antibiotics while breastfeeding. To reduce the impact of maternal diet or genetics, an effort was made to obtain samples from women of the same ethnicity within a country. Research ethics approval was obtained from the local ethics committees of participating institutions: Human Research Ethics Committee of The University of Western Australia, Human Research Ethics Committee of the Princess Margaret Hospital, Committee on Human Research of Chiba University, Institutional Review Board at Henry Ford Health System, Norwegian Regional Committees for Medical and Health Research Ethics, and University of Cape Town Human Research Ethical Committee.

### Maternal Atopic Status

Maternal atopic status was defined according to maternal report of having asthma, eczema or atopic dermatitis, or a pet, environmental or food allergy (Norwegian, South African women), or atopic sensitization on the basis of at least one blood allergen-specific IgE level ≥ 0.35 kU/L (US women) to house dust mite, dog, cat, Timothy grass, ragweed, *Alternaria alternata*, egg, or German cockroach, or at least one blood allergen-specific IgE level ≥ 0.7 kU/L (Japanese women) to house dust mite, cat or Japanese cedar, or at least one positive skin prick test (Australian women) to house dust mite, dog, cat, Timothy grass, Japanese cedar where applicable, ragweed, *Alternaria alternata*, egg, or German cockroach. Australian and Japanese atopic women also had a physician-diagnosed history of asthma, eczema or atopic dermatitis concurrent with atopic sensitization.

### Human Milk Sample Collection

HM samples were collected 1 month after birth, a time point at which the composition of human milk is thought to stabilize (28). Participants were given written and oral instructions to standardize self-collection of samples. Prior to collection, nipples and mammary areola were cleaned with soap and sterile water, and for the samples from South Africa, additional cleaning was performed with chlorhexidine to reduce contamination by skin microbes. Human milk samples were expressed manually or with an electric breast pump into a sterile tube. Australian samples from non-atopic women (2015) and Norwegian samples (2002) were stored at −20°C, Australian samples from atopic women (2002) and samples from US women (2003) were stored at −80°C. The samples from Japanese women (2010) were initially stored at −80°C before being moved to −30°C. Samples were shipped on dry ice to The Metabolomics Innovation Center, Edmonton, Canada for processing in 2015.

### NMR Analysis

Milk metabolite levels were determined by NMR because of its high reproducibility and coverage of a large range of metabolites. Samples were analyzed as previously reported by Gay et al. (5). Briefly, samples were thawed on ice, mixed thoroughly, and then filtered to remove residual lipids and proteins using a 3-kDa cutoff spin filter at 10,000 × *g* for 15 min at 4°C. Three hundred fifty microliter of filtrate was transferred to a clean tube, and 70 μL of D_2_O and 60 μL of standard buffer solution (585 mM NaHPO_4_ (pH 7.0), 11.667 mM disodium-2,2-dimethyl-2-silapentane-5-sulfonate (DSS), and 0.47% NaN_3_ in H_2_O) were added. Samples were then transferred to regular NMR tubes for subsequent NMR spectral analysis. All 1H-NMR spectra were collected on a Varian 500 MHz Inova spectrometer equipped with a 5-mm HCN Z-gradient pulsed-field gradient cryogenic probe. 1H-NMR spectra were acquired at 25°C using the first transient of the Varian tnnoesy pulse sequence (chosen for its high degree of selective water suppression and quantitative accuracy of resonances around the solvent). Water suppression pulses were calibrated to achieve a bandwidth of 80 G. Spectra were collected with 128 transient and 8 steady-state scans using a 4-s acquisition time (48,000 complex points) and a 1-s recycle delay. Quality control (QC) mixtures consisting of 4 metabolites at 1 mM were analyzed for every 20 to 25 samples, and a relative standard deviation of <2% was observed. Prior to spectral analysis, all free induction decays were zero-filled to 64,000 data points and line broadened to 0.5 Hz. The methyl singlet produced by a known quantity of DSS was used as an internal standard for chemical shift referencing (set to 0 ppm) and for quantification. All 1H-NMR spectra were processed and analyzed using the Chenomx NMR Suite Professional software package version 8.1. Typically, 90% of visible peaks were assigned to a compound, and more than 90% of the spectral area could be routinely fit using the Chenomx spectral analysis software. Most of the visible peaks were annotated with a compound name and expressed as μmol/L. The limit of detection for these compound was 5-6 μmol/L.

### Statistical Analysis

Statistical analyses were carried out using R studio 1.1.414 (Rstudio Inc., Boston, MA, USA) with package nlme for linear mixed models to test statistically significant differences between HM metabolites by atopic status within each country and by country within atopic status. The Tukey–Kramer test was used to adjust for multiple comparisons. Differences were considered to be statistically significant if *p* < 0.05. Partial Least Squared Discriminant Analysis (PLS-DA) plots were created using an Excel add-in Multibase 2015 package (Numerical Dynamics, Japan) to maximize the separation of HM clusters by maternal atopic status. Correlations between SCFAs were determined using Spearman's rank correlation.

## Results

Of the 109 participating women, 43% were classified as atopic (Table 1). There was generally an even distribution of atopic/non-atopic mothers between the cohorts, except for South Africa, where only non-atopic women were sampled. Overall, 69% of participants were Caucasian. The majority of South African women were of mixed race, 39% of the US cohort were African American, and most of the Australian and Norwegian cohorts were of Caucasian ancestry. Cohorts were comparable with respect to maternal age, parity and pre-pregnancy BMI; Japanese women had the lowest BMI, whereas Australian women were the oldest and had the lowest parity. All but one woman had delivered vaginally. Only nine women reported taking antibiotics and use was during early pregnancy or delivery.

**Table 1:** Characteristics of the cohort (*n* = 109).

	**Australia**	**Japan**	**Norway**	**South Africa**	**USA**
	**(*n* = 29)**	**(*n* = 12)**	**(*n* = 40)**	**(*n* = 10)**	**(*n* = 18)**
Maternal atopy	21 (72%)	6 (50%)	9 (23%)	0 (0%)	11 (61%)
Maternal race					
Caucasian	28 (100%)	0 (0%)	34 (85%)	2 (20%)	11 (61%)
Asian	0 (0%)	12 (100%)	0 (0%)	0 (0%)	0 (0%)
Black	0 (0%)	0 (0%)	0 (0%)	2 (20%)	7 (39%)
Mixed race	0 (0%)	0 (0%)	0 (0%)	6 (60%)	0 (0%)
Other race	0 (0%)	0 (0%)	6 (15%)	0 (0%)	0 (0%)
Maternal age (years)	33.8 ± 5.2	24.6 ± 5.5	29.4 ± 5.2	29.8 ± 4.8	29.6 ± 4.4
Maternal parity	1.3 ± 0.5	1.7 ± 1.0	1.5 ± 0.5	2.0 ± 0.9	2.2 ± 1.2
Maternal pre-pregnancy BMI		20.7 ± 2.5	28.1 ± 6.6	25.0 ± 2.9	27.2 ± 5.6
Maternal antibiotics	4 (14%)^*^	0 (0%)	5 (13%)^∧^	0 (0%)	0 (0%)
Cesarean delivery	1 (4%)	0 (0%)	0 (0%)	0 (0%)	0 (0%)
Male infant	13 (46%)	6 (50%)	25 (63%)	5 (50%)	6 (33%)

*Values are reported as n (percent) or mean ± SD*.

*Blank cells represent missing data*.

* *One case of intrapartum Cefazolin for cesarean delivery, two cases of intrapartum penicillin for Group B Streptococcus, one case of intrapartum antibiotics with no class or reason recorded*.

∧ *All exposures were in early pregnancy. Class of antibiotic was not recorded*.

Full metabolomic data from this cohort have previously been reported (5). In brief, HM samples from atopic and non-atopic mothers clustered separately (Supplementary Figure 1). For the purposes of this study, we have focused on the SCFAs in HM, which have not been previously reported in this cohort.

### Human Milk Contains Short Chain Fatty Acids

All samples contained detectable levels of acetate, butyrate, and formate (Table 2). Propionate and valerate were not detected in any of the samples. Butyrate was the most abundant SCFA in these samples (median level of 95.6 μmol/L), followed by acetate (median level of 46.8 μmol/L), and formate (median level of 43.7 μmol/L). There were statistically significant positive correlations between acetate and butyrate (rho = 0.55, *p* = 6.66 × 10^−10^) and acetate and formate levels (rho = 0.33, *p* = 0.0006). The SCFA intermediates pyruvate, lactate, and succinate were also detected (Supplementary Table 1).

**Table 2:** Levels of short chain fatty acids detected in 109 human milk samples taken at 1 month postpartum.

	**Formate**	**Acetate**	**Propionate**	**Butyrate**	**Iso-butyrate**	**Valerate**	**Iso-valerate**
	**(C1:0)**	**(C2:0)**	**(C3:0)**	**(C4:0)**	**(C5:0)**	**(C5:0)**	**(C5:0)**
Prevalence	100%	100%	0%	100%	0%	0%	0%
Median	43.7	46.8	-	95.6	-	-	-
Minimum	15.2	13.5	-	4.8	-	-	-
Maximum	4960.3	4307.7	-	409.5	-	-	-

*Values are reported as % prevalence or μmol/L*.

### Human Milk Short Chain Fatty Acids Differ Geographically and by Maternal Atopic Status

**Figure 1:**
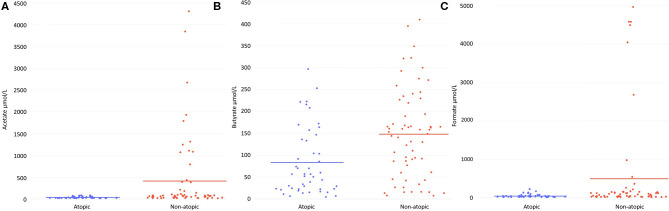
Human milk levels (μmol/L) from non-atopic (*n* = 62) and atopic (*n* = 47) mothers of **(A)** acetate, **(B)** butyrate and **(C)** formate. Lines indicate mean values.

HM from atopic women had significantly lower levels of the SCFAs acetate (*p* = 0.02) and butyrate (*p* = 0.001) than that of non-atopic women (Figure 1). Median levels of these SCFAs in atopic women were approximately half that of their non-atopic counterparts (57% lower for acetate, 62% lower for butyrate). Only for Australian women, of whom 100% were Caucasian, were acetate and butyrate levels significantly lower in those with vs. those without atopy (*p* = 0.009 and *p* = 0.002, respectively). Acetate levels were lower in atopic vs. non-atopic Norwegian (85% Caucasian, *p* = 0.009). The reduction in HM acetate levels with atopy in Japanese women (100% Asian) did not reach statistical significance (*p* = 0.2). Among women from the US (61% Caucasian), HM acetate levels were higher with atopic than non-atopic disease (*p* = 0.02). This difference was driven by samples from atopic Black women as when the comparison was restricted to Caucasian women, differences were no longer statistically significant. HM formate levels were also lower in atopic than non-atopic women (45% lower, *p* = 0.056) (Figure 1); this difference was statistically significant within Australian women (*p* < 0.0001) and within Norwegian women (*p* = 0.009). Overall, there were no differences in HM levels of the SCFA intermediates pyruvate, lactate, and succinate between atopic and non-atopic women (Supplementary Table 1). However, the HM of Australian women with atopy also had higher levels of lactate (*p* = 0.01) and pyruvate (*p* < 0.0001), and lower levels of succinate (*p* = 0.003) than of women without atopy.

**Figure 2:**
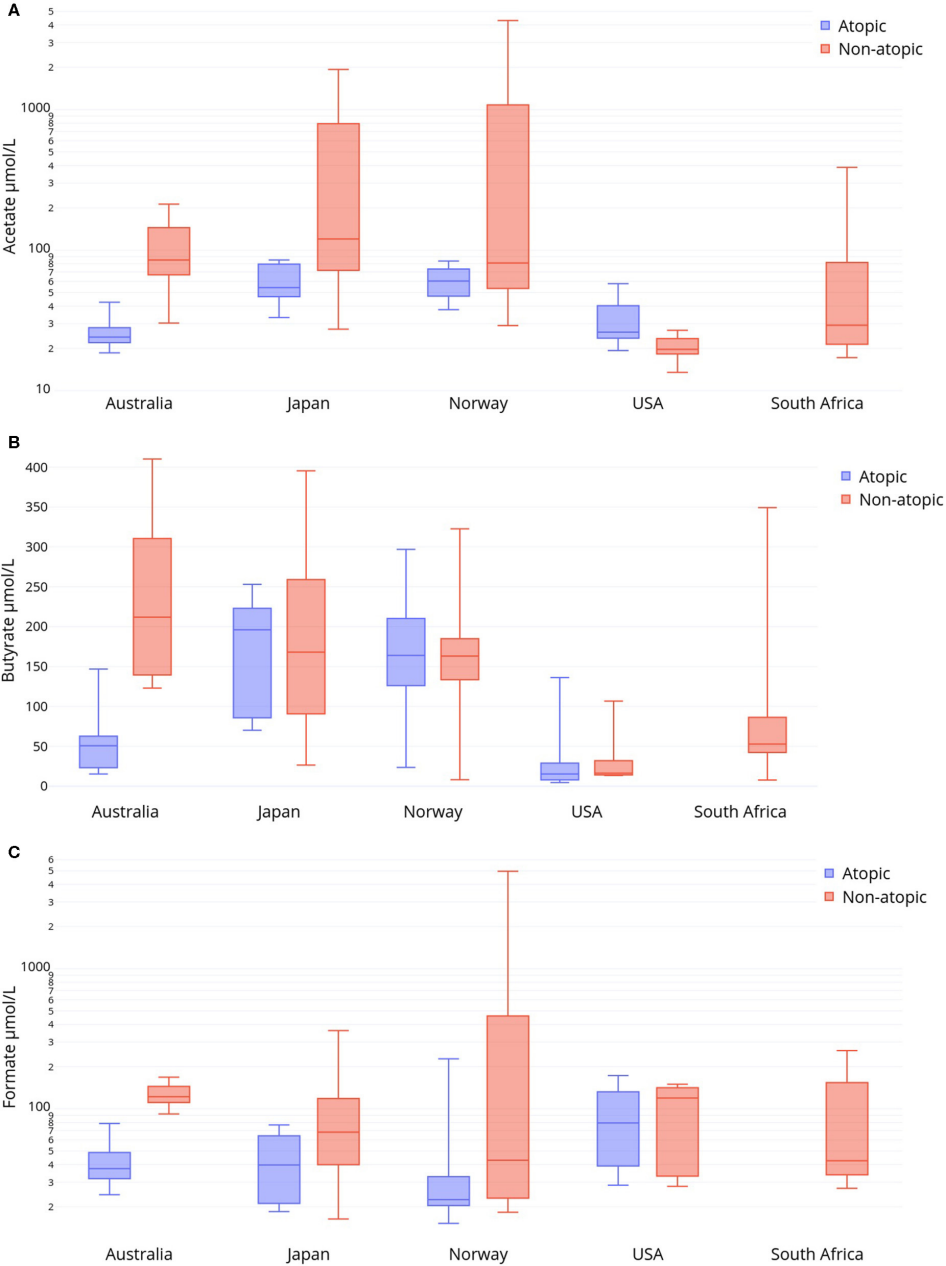
Human milk levels (μmol/L) from non-atopic and atopic mothers in five international sites of **(A)** acetate, **(B)** butyrate and **(C)** formate. Boxes represent median and IQR, whiskers represent range.

Variations in HM SCFAs levels were also seen between women of the same atopic status living in different countries (Figure 2, Supplementary Table 2). As tested by mixed linear models, HM levels of butyrate were significantly lower in non-atopic US women compared to those living in Australia, Norway, or Japan (*p* = 0.001, *p* = 0.004, and *p* = 0.02, respectively) but not South Africa. HM butyrate levels were reduced in non-atopic South African women compared to non-atopic Australian women (*p* = 0.01). We conducted a sensitivity analysis that subdivided non-atopic women in the US by race into Black or Caucasian. Only among non-atopic Caucasian women from the US did butyrate levels remain significantly lower than among women in the above comparison.

Atopic women did not differ in their milk acetate or butyrate profiles if they lived in the US vs. Australia, or in Norway vs. Japan. HM from atopic Australian and US women had significantly lower levels of acetate and butyrate that that of atopic Norwegian and Japanese women (*p* < 0.001). HM from atopic Australian women also had lower levels of formate compared to that of US women (*p* = 0.02). No other differences in milk SCFA levels between countries were observed within either non-atopic or atopic women. In a sensitivity analysis that subdivided atopic women in the US by race, atopic Black or Caucasian women from the US continued to differ from Norwegian and Japanese atopic women in terms of lower acetate and butyrate levels in HM. Restricting the US-Australia comparison to atopic Caucasian women did not alter the lack of statistical difference for HM acetate or butyrate.

Levels of SCFA intermediate products also differed by country (Supplementary Table 2). Lactate levels were significantly higher in HM from non-atopic Japanese women compared to HM from other non-atopic women (Norway *p* = 0.003, South Africa *p* = 0.006, Australia *p* = 0.008, USA *p* = 0.01). Similarly, lactate was elevated in HM from atopic Japanese mothers compared to atopic Norwegian mothers (*p* = 0.01). HM from non-atopic Japanese and South African mothers had significantly higher levels of pyruvate compared to non-atopic Australian and Norwegian mothers (Japan v. Australia *p* = 0.004; Japan v. Norway *p* = 0.008; South Africa v. Australia *p* = 0.02; South Africa v. Norway *p* = 0.04). Finally, atopic Norwegian women had significantly higher levels of HM succinate compared to mothers from other countries (*p* < 0.001).

## Discussion

Here we report that HM contains detectable levels of SCFAs acetate, butyrate, and formate at 1 month postpartum. Collectively, HM levels of acetate and butyrate were significantly reduced in atopic women. This trend was retained for HM acetate in atopic women in Australia, Norway and Japan but not the US. Only among Australian women were HM formate and butyrate levels lower with atopic disease. SCFAs have been shown to be provide protection from allergy and atopy in mice, particularly through their effects on regulatory T cell and dendritic cell biology (8, 9, 16). Higher relative levels of fecal acetate during pregnancy have been associated with reduced risk for hay fever, asthma and wheeze in the offspring of mothers with a history of atopy (15). In their study, fecal acetate levels were higher in mothers of breastfed infants. The ability of SCFAs to inhibit histone deacetylases suggests a role for HM-derived SCFAs in the epigenetic regulation of immune function and postnatal programming of atopy in breastfed offspring. Reduced levels of SCFAs in the HM of atopic women may therefore play a role in the intergenerational transmission of atopic disorders. Indeed, recent data demonstrate that low HM bacterial richness is associated with atopy development in early life (29). Gomez-Gallergo et al. reported country differences in HM SCFA and their correlations with HM microbiota (26). We extend those findings by identifying maternal atopic status as a possible source of variation in HM SCFA.

The reduced levels of acetate in milk from atopic mothers may have other physiological consequences for breastfed infants. In cows, acetate is the major substrate of *de novo* fat synthesis in milk (30). It is unclear whether this is also true for humans (31), but HM acetate levels are found to be weakly correlated with HM fat concentrations (22). In general, breastfeeding is associated with reduced infant adiposity, and gut acetate levels are highest in exclusively breastfed infants (32, 33). SCFAs are involved in several biologic pathways that prevent overweight, including appetite suppression and promotion of fat oxidation over fat synthesis (7). Indeed, HM acetate levels are reported to be negatively associated with infant skinfold thickness (22). Maternal atopic status appears to over-ride the protective actions of prenatal anti-inflammatory cytokines against overweight development in offspring (34). Our study suggests that maternal atopic status may also reduce the availability of HM SCFAs to regulate fat metabolism in the breastfed infant. Acetate and butyrate are also involved in the production of long-chain fatty acids (31). However, contrary to our findings, HM long-chain fatty acid levels do not appear to differ by atopic status (35–37).

HM SCFA levels also varied between our cohorts. This is unsurprising given that the early life gut, adult gut, and HM microbiomes vary geographically (38–41). These metabolites are also likely influenced by regional differences in diet that feed the gut microbiota toward enrichment with Bacteroidetes species in US/European populations. Since we did not collect maternal fecal or HM samples for bacterial profiling, we are unable to link alterations in HM SCFA profiles with specific members of the bacterial community. Gronlund et al. reported reduced bifidobacterial abundance in HM and in the gut microbiota of breastfed infants if mothers had atopic disease (42). Higher bifidobacterial abundance by 3 months of age, followed by an earlier switch to increasing abundance of butyrate-producing bacteria, has been found to be protective against later risk of atopy (43). Recently, *Bifidobacterium*, a key acetate-producing genus, was found to be less abundant in the stool of breastfed infants in the US vs. several African countries (41). Additionally, HM from mothers of US infants exhibited much lower overall bacterial diversity (41). While *Bifidobacterium* spp. chiefly produce acetate, they form symbiotic relationships with butyrate-producers such as *Eubacterium* (44). In HM, acetate and butyrate levels are positively correlated (22). It is thus interesting to note the exceptionally low levels of butyrate in HM from non-atopic US mothers of Caucasian ancestry.

Acetate, butyrate, and formate have been found in HM of women worldwide (20–26). The levels of SCFAs reported here are in line with those recently reported by Prentice et al. as determined by NMR and GC-MS (at 1-2 months postpartum), and by Wu et al. (across lactation) and Smilowitz et al. (at 3 months) by NMR (20–22). We also confirm the positive correlations of HM acetate with butyrate and formate reported by Prentice et al. The failure by us and others to detect propionate in HM is curious. Presumably, SCFAs, which are produced in the gut, enter HM from the maternal circulation. Thornburn et al. reported that the three most abundantly produced SCFAs in humans (acetate, butyrate, and propionate) were approximately equal in concentration in the sera of pregnant women (median levels 51.4 μmol/L for acetate, 37.1 μmol/L for propionate, and 35.6 μmol/L for butyrate) (8). SCFAs present in HM may be produced by the resident HM microbiota; however, evidence for this possibility is currently lacking. Regardless, the presence of SCFAs in HM likely has important consequences for the developing infant. Endogenous production of SCFAs is low in early infancy (45). Maternally provided SCFAs may, therefore, supplement breastfed infants during the early periods of gut microbiome immaturity.

A major strength of our study is the use of multiple cohorts from around the world. However, this also means that samples were not uniformly collected and stored. Lack of standardized collection by time of day is not an issue for our comparison since there is no evidence for diurnal variation in HM SCFAs (21). On the other hand, some SCFAs are sensitive to storage temperatures higher than −80°C, the temperature at which SCFAs are highly stable for up to 2 months (46). Slight increases to levels of HM butyrate (4 μmol/L) are initially seen after short periods of storage at −20°C compared to storage at −80°C (21), followed by modest declines in butyrate with longer HM storage times at −20°C for up to 16 years (22). Unfortunately, no studies have compared long term storage at −20°C to −80°C. While variation in storage conditions may be an unavoidable limitation of our study, it is unlikely to explain the much lower levels of HM butyrate observed in the Australian atopic samples (stored at −80°C) or to explain within country differences or between country similarities. More importantly, the very large difference in milk butyrate between our Australian cohorts (161 μmol/L lower levels in atopic women) is in the opposite direction to the above stability findings since atopic samples were stored immediately at −80°C, whereas the more recently-collected non-atopic samples were stored at −20°C. Our non-standard definition of “atopy” across cohorts is also a major limitation of this comparison, although similar trends were observed for HM from Norway and Japan despite the absence of serum IgE testing in Norwegian women. Other limitations include not having samples from atopic women from South Africa, and lacking balance in atopy status and number of participants per country. Finally, data were not available for all cohorts on maternal parity, body-mass index or socioeconomic status, but these characteristics have not been found to be correlated with HM SCFA levels (22). On the other hand, this study would have benefited from information on maternal diet, which may have strengthened similar findings by atopic status in two countries with high fermented food intake—Norway and Japan.

## Conclusion

Our findings suggest that HM SCFA levels may vary by maternal atopic status and country of residence, a finding that could not be attributed to race. Despite sharing Caucasian ancestry, HM SCFA profiles for atopic women differed in Norway vs. the US or Australia. On the other hand, similar HM SCFA profiles by atopic status were seen in Norway and Japan. Lower levels of HM SCFAs have the potential to alter immune programming and fat metabolism in the breastfed offspring of women. This has implications for non-atopic women as well. In our study, this singled out women in the US who had the lowest levels of HM acetate and butyrate compared to non-atopic women in other countries.

## Data Availability Statement

The datasets generated for this study are available on request to the corresponding author.

## Ethics Statement

The studies involving human participants were reviewed and approved by Human Research Ethics Committee of The University of Western Australia, Human Research Ethics Committee of the Princess Margaret Hospital, Committee on Human Research of Chiba University, Institutional Review Board at Henry Ford Health System, Norwegian Regional Committees for Medical and Health Research Ethics, and University of Cape Town Human Research Ethical Committee. The patients/participants provided their written informed consent to participate in this study.

## Author Contributions

PK performed the NMR analysis. LS drafted the manuscript and contributed to data analysis and visualization. MG contributed to data analysis and visualization. ET, ME, CJ, GW, NS, DC, SP, DM, DG, and AK oversaw recruitment, sample collection, storage, and funding for their respective cohorts. All authors reviewed and critically edited the manuscript.

## Conflict of Interest

All authors declare that the research was conducted in the absence of any commercial or financial relationships that could be construed as a potential conflict of interest.

## Supplementary Material

Supplementary Figure 1 Partial Least Square Discriminant Analysis (PLS-DA) loading plot **(Left)** and scatterplot **(Right)** of human milk metabolites from 109 women in various countries. The score plot shows separation based on maternal atopic status. The loading plot shows the milk metabolites that influence the separation based on maternal atopic status.

Supplementary Table 1 Levels of short chain fatty acid intermediates detected in human milk samples from atopic (*n* = 47) and non-atopic (*n* = 62) women at one month postpartum. Values are reported as % prevalence or μmol/L.

Supplementary Table 2 Mean levels of short chain fatty acid and intermediates detected in human milk samples from atopic and non-atopic women at one month postpartum in five international cohorts. Values are reported as μmol/L.
