# Supplementary figures and images for: Human Milk From Atopic Mothers Has Lower Levels of Short Chain Fatty Acids
Source: Front Immunol. 2020 Jul 21;11:1427. doi: 10.3389/fimmu.2020.01427 (PMC7396598; doi:10.3389/fimmu.2020.01427)

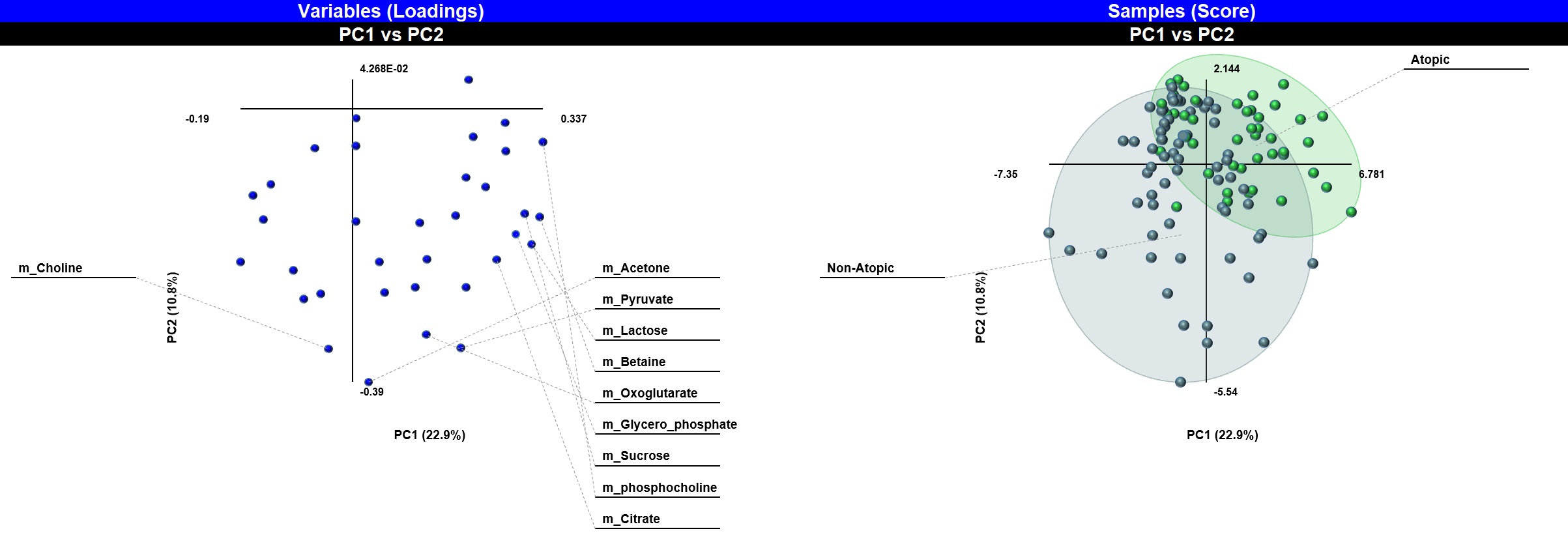

Supplement: Supplementary Figure 1 — Partial Least Square Discriminant Analysis (PLS-DA) loading plot (Left) and scatterplot (Right) of human milk metabolites from 109 women in various countries. The score plot shows separation based on maternal atopic status. The loading plot shows the milk metabolites that influence the separation based on maternal atopic status. [file Image_1.JPEG]
