# Supplementary material for: Human Milk From Atopic Mothers Has Lower Levels of Short Chain Fatty Acids
Source: Front Immunol. 2020 Jul 21;11:1427. doi: 10.3389/fimmu.2020.01427 (PMC7396598; doi:10.3389/fimmu.2020.01427)
Supplement: Supplementary Table 1 — Levels of short chain fatty acid intermediates detected in human milk samples from atopic (n = 47) and non-atopic (n = 62) women at one month postpartum. Values are reported as % prevalence or μmol/L. [file Table_1.DOCX]

**SUPPLEMENTARY TABLE 1:** Levels of short chain fatty acid intermediates detected in human milk samples from atopic (*n* = 47) and healthy (*n* = 62) women at one month postpartum. Values are reported as % prevalence or µmol/L.

|  | **Lactate** | | **Pyruvate** | | **Succinate** | |
| --- | --- | --- | --- | --- | --- | --- |
|  | **Atopic** | **Healthy** | **Atopic** | **Healthy** | **Atopic** | **Healthy** |
| Prevalence | 100% | 100% | 100% | 100% | 100% | 100% |
| Median | 170.9 | 170.9 | 25.7 | 10.2 | 30.3 | 46.7 |
| Minimum | 48.2 | 35.5 | 0.7 | 1.3 | 10.7 | 8.6 |
| Maximum | 780.1 | 14329.3 | 89.3 | 186.3 | 117.8 | 2928.9 |

**SUPPLEMENTARY TABLE 2:** Mean levels of short chain fatty acid and intermediates detected in human milk samples from atopic and healthy women at one month postpartum in five international cohorts. Values are reported as µmol/L.

|  | **Australia** | | **Japan** | | **Norway** | | **South Africa** | **USA** | |
| --- | --- | --- | --- | --- | --- | --- | --- | --- | --- |
|  | **Atopic** | **Non-atopic** | **Atopic** | **Non-atopic** | **Atopic** | **Non-atopic** | **Non-atopic** | **Atopic** | **Non-atopic** |
| Acetate | 25.4 | 104.5 | 58.8 | 510.7 | 60.1 | 651.7 | 102.7 | 32.5 | 20.4 |
| Butyrate | 51.5 | 232.1 | 170.6 | 184.6 | 164.1 | 162.5 | 97.2 | 27.8 | 32.4 |
| Formate | 39.9 | 127.1 | 43.4 | 112.2 | 47.6 | 902.0 | 83.4 | 86.6 | 91.9 |
| Lactate | 209.3 | 125.7 | 287.7 | 3215 | 97.9 | 443.5 | 213.7 | 196.9 | 137.8 |
| Pyruvate | 48.7 | 6.3 | 21.2 | 68.6 | 9.7 | 20 | 53.3 | 30.4 | 31.7 |
| Succinate | 30.1 | 49.3 | 28.7 | 47.9 | 76.2 | 210.8 | 48.3 | 37.2 | 21.2 |
